# The Effect of Prognostic Factors on Long-Term Protection in Breast Cancer

**DOI:** 10.7759/cureus.8878

**Published:** 2020-06-28

**Authors:** Taner Evcimik, Mahmut Said Degerli, Tayfun Bilgic

**Affiliations:** 1 General Surgery, Acibadem Fulya Hospital, Istanbul, TUR; 2 General Surgery, Haseki Traning and Research Hospital, Istanbul, TUR; 3 Medical Service and Techniques, Nisantasi University Vocational High School, Istanbul, TUR

**Keywords:** cancer, er, pr, cerb b2, progesterone receptor, estrogen receptor, survival rate

## Abstract

Objectıves

Axillary lymph node involvement is considered to be one of the most important factors in the staging and survival of breast cancer. Recurrences in women with a negative axillary condition detected as a result of long patient follow-up have revealed the importance of other prognostic factors and many studies have begun. The aim of this study is to evaluate breast cancer patients and to investigate other factors affecting breast cancer.

Materials and methods

Patients with breast cancer who were operated in our clinic between January 2005 and June 2007 were included in the study. Demographic characteristics, tumor size, lymph node involvement, grade, histological type, the status of estrogen and progesterone receptors (ER and PR), type of surgery performed, and cerb-B2 receptor status were recorded retrospectively.

Results

The mortality rate of the ER (+) PR (+) group was significantly lower than that of the ER (-) PR (-) group (p<0.05). There was no statistically significant difference between the ER (+) PR (+) cases and the mortality rates of any positive cases (p>0.05). There was a statistically significant difference between survival rates according to cerb-B2 status (p<0.01); cerb-B2 was positive in all patients who died. In cerb-B2 positive cases, there was a statistically significant difference between survival rates according to the tumor stages (p <0.05).

Conclusion

As a result; preoperative and postoperative staging of all breast cancer patients who applied to surgery clinics should be performed. Prognostic factors should be determined and patients should be directed to post-surgical treatment according to this information. Axillary lymph involvement, number, tumor size, estrogen receptor, progesterone receptor, and cerb-B2 status are safe markers that can be used to determine prognosis in our series.

## Introduction

Breast cancer is the most common malignant tumor among women worldwide. In addition, the most common cause of cancer-related deaths in women is breast cancer [1-2]. Recent advances in the early diagnosis and treatment of breast cancer have led to significant advances in the overall survival and disease-free survival of breast cancer [3-4].

Axillary lymphocyte involvement is the most important factor in the staging and survival of breast cancer [5]. Recurrences in breast cancer patients with negative axilla involvement after long patient follow-up revealed the importance of other prognostic factors. Numerous studies have begun on this subject (c-erbB-2, p53, cathepsin D, and so on). Although new genomic studies provide a comprehensive catalog of genes that accumulate somatic point mutations and small additions (indels) in estrogen receptor-positive (ER+) breast cancer, significant uncertainties remain regarding how these newly discovered mutations relate to disease outcomes [6-8].

The aim of this study was to evaluate breast cancer patients who applied for breast cancer and to investigate other factors affecting survival in the long term.

## Materials and methods

Patients with breast cancer who were operated in our clinic between January 2005 and June 2007 were included in the study. Demographic characteristics, tumor size, lymph node metastasis status, grade, histological type, estrogen and progesterone receptor status, type of surgery performed, and cerb-B2 overexpression were recorded retrospectively.

The survival and metastasis information of seven out of 71 patients were excluded from the study. The data of 64 patients were evaluated. The survival rates of the patients according to their pathological stages, axilla status (N1, 2, 3), estrogen receptors (ER), progesterone receptor (PR), and cerb-B2 status were investigated and the factors affecting the survival rate were investigated statistically.

For the statistical analysis, the SPSS (Statistical Package for Social Sciences) for Windows 15.0 program (SPSS Inc., Chicago, IL) was used. In the evaluation of the data, descriptive statistical methods (mean, standard deviation), as well as the qualitative data, were compared by the chi-square test and the Fisher methods exact chi-square test; The significance test of the difference between the two percentages was used. The Kaplan Meier and log-rank tests were used in survival evaluations. The results were evaluated at the 95% confidence interval and the p<0.05 significance level.

## Results

The demographic and tumor characteristics of 64 patients included in the study are shown in Table 1. A total of three (4.7%) patients had local recurrence and six (9.3%) had systemic metastasis. Mortality was detected in three (4.7%) cases during the follow-up period. The mean follow-up period was 22.66 ± 9.69 (7-38) months. When the survival rates, according to the stages of the patients were examined, a statistically significant difference was observed between the stages (p <0.01). As the stage increases, mortality rates increase.

**Table 1:** Demographic characteristics of patients ER: estrogen receptor; IDC: invasive ductal carcinoma; ILC: invasive lobular carcinoma; DCIS: ductal carcinoma in situ; LCIS: lobular carcinoma in situ

	%
Average age	52.34±10.72 (32-83)
Mean lymph node resection	11.79±4.47 (2-25)
Mean number of metastatic lymph nodes	2.70±3.63 (0-15)
Mean ER level	54.09±34.22 (0-95)
Surgical	
Breast protection surgery	21.9
Mastectomy	78.1
Tumor type	
IDC	75
ILC	18.7
DCIS	4.7
LCIS	1.6
Grade	
1	9.4
2	78.1
3	12.5
T stage	
T1	37.5
T2	56.3
T3	6.2
Stage	
1	15.6
2A	42.2
2B	15.6
3A	26.6
ER	
Positive	78.1
Negative	21.9
PR	
Positive	85.9
Negative	14.1
Cerb-B2	
Positive	42.2
Negative	57.8

The mortality rates were found to be 4/46 (8.7%) in ER(+) and PR(+) cases; 2/5 (40%) in ER(-) and PR(-) cases; 1/13 (7.7%) in ER(+) or PR(+) cases. The mortality rate of the ER (+) PR (+) group was significantly lower than the ER (-) PR (-) group (p<0.05). There was no statistically significant difference between the ER (+) PR (+) cases and the death rates according to the ER(+) or PR(+) group (p>0.05). Although the mortality rates of ER (-) PR (-) cases were found to be higher than the mortality rates in any of the positive cases, there was no statistically significant difference in the number of cases in the groups (p>0.05). The survival distribution of the patients according to the stages is given in Table 2.

**Table 2:** Survival distribution in each stage according to ER and PR status ER: estrogen receptor; PR: progesterone receptor

	ER (+) PR (+)	ER (-) PR (-)	ER (+) or PR(+)
Stage 1	7/0	0	3/0
Stage 2A	22/0	0	5/0
Stage 2B	7/0	1/1	2/0
Stage 3A	10/4	4/1	3/1

There was a statistically significant difference between the survival rates of CerbB2 (p <0.01); Cerb2 was positive in all patients who died. There was a statistically significant difference between survival rates according to the stages in Cerb B2 positive cases (p<0.05); As the stage increases, the mortality rate increases, and the survival rate decreases. In the Cerb B2 negative cases, there was no statistical evaluation because no death was observed.

**Figure 1:**
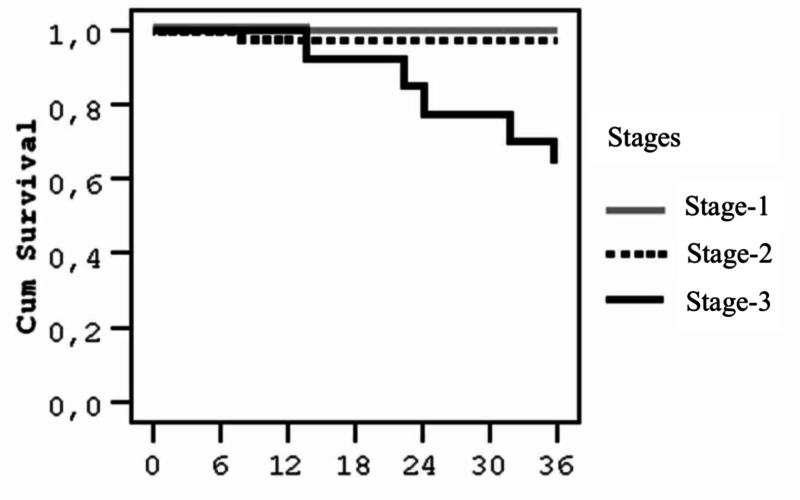
Survival analysis by stage

The study was started with 64 cases. Of the cases included in our study, seven patients had death or metastasis within the follow-up period and the last death time was 36 months. The survival rate corresponding to this time is 71.15%. The standard error is 1.06. Survival rates according to stages are given in Table 3. When compared to the log-rank test, survival was found to be lower than that of Stage 1 (p=0.002; p<0.01) (Figure 1).

**Table 3:** Survival status according to stages

Stages	Number of patients	Number of mortality	Survival rate %
Stage 1	10	0	100
Stage 2	37	1	97.3
Stage 3	17	6	64.7

The mortality was 6/36 cases with axillary involvement patients, and in these patients, the survival rate was 83.3% during the follow-up period. The mortality was 1/28 in patients who have no axillary involvement and the survival rate was 96.4% during the follow-up period. There was no statistically significant difference between survival according to axillary involvement (p=0.234; p>0.05).

There was no mortality in 15 patients who have one to three axillary lymph node involvement in follow-up periods. There were 21 patients who have four or more axillary lymph node involvement and six of them have died during the follow-up period. The survival rate was 71.4% in these patients. The number of axillary lymph node involvement was found to have a statistically significant effect on survival rates (p=0.054; p>0.05).

We detected seven cases with metastasis in the analyzed cohort of breast cancer patients. Those cases included six patients at Stage 3A and one patient at Stage 2B. Four or more axillary lymph nodes were involved in six patients, and there was one patient who had no axillary involvement. In four cases, both ER and PR were positive, while in two cases, both were negative. While the cerb-B2 score was positive in four cases, cerb-B2 was negative in three cases. Local recurrence developed in three cases in the 2A Stage. All of the patients had the involvement of three lymph nodes, all of which were positive for ER and PR, and two cases were cerb-B2 positive.

## Discussion

The clinical behavior of breast cancer is characterized by a long natural course and heterogeneity. The clinical history of breast cancer is also variable and the survival time of untreated patients with the same clinical stage of the disease varies from several months to decades [1].

This study provides information on the effect of somatic mutations in ER (+) breast cancer with long-term follow-up and controlled treatment, providing new data on the sample. So far, many effective treatment models for breast cancer have been developed. However, each treatment strategy has been effective only for some individuals with breast cancer. Therefore, although we have predicted the natural course of the disease, it is very important to identify prognostic markers that allow us to determine the optimal treatment strategy and assess the future of the disease.

Breast cancer is not a single disease, with different courses and prognoses, different pathological processes should be evaluated as a combination of different pathological processes is becoming increasingly widespread. Long-term survival is associated not only with an early diagnosis but also with the biological behavior of the tumor and the potential for malignancy. The definition of recovery in breast cancer is also complex [2]. The most commonly used concept is statistical improvement. All of the studies evaluating the statistical improvement in breast cancer patients showed an ongoing mortality risk [3-4]. In these studies, the survival curve of breast cancer patients never becomes parallel to the normal population, and even after 25-40 years, the mortality risk continues to increase. In this study, we use the deoxyribonucleic acid (DNA) from the archive material to investigate the prognostic effects encoded by the mutational landscape of breast cancer. We have successfully used properly treated patient samples. We were able to confirm our prospective hypothesis from our previous studies.

Axillary nodal involvement is highly correlated with prognosis. In patients with histologically negative axillary lymphocyte involvement, the survival rate is significantly greater than in patients with axilla lymphocyte involvement. In addition, the prognosis of those with one to three lymph node involvement is better than those with four or more lymph node involvement [5]. In our study, there was no statistically significant difference between the axilla status and survival. The survival rate was 96.4% in patients without axilla involvement and 83.3% in patients with axillary positivity. In addition, in patients with one to three lymph nodes positive, survival is 100%. It was found to be 71.4% in patients with four or more positive lymph nodes, and these results were close to statistical significance (p=0.054).

The results of the studies are used to determine the steroid receptor status treatment decisions in breast cancer. Knight et al. showed that in 1977, ER had an independent prognostic significance in terms of early recurrence in early breast cancer [6]. Longer follow-up studies have shown that survival in ER (+) cases is longer than ER (-) cases [7]. Generally, ER has a strong predictive value for disease-free survival; PR is associated with overall survival because it is indicative of a better response to endocrine therapy in the event of disease recurrence. ER positivity, survival, and disease-free survival were higher in differentiated tumors [8]. In addition to studies that have no relationship between ER and axillary lymph node involvement [9], there are studies showing that there is a relationship between ER status and axillary lymph node metastasis in T1 tumors [10]. In our study, the mortality rate in cases with ER (+) - PR (+) and ER-PR were positive; ER (-) - PR (-) was found to be statistically significant (p<0.05). These results - the fact that ER and/or PR are positive is a significant prognostic factor and these patients have longer survival.

Gene amplification was measured in the first studies investigating the prognostic significance of cerb-B2. Survival was found to be worse in patients with axillary positive and cerb-B2 amplification [11]. In our study, the rate of cerb-B2 positivity was 42.2%. Although it is emphasized that there is a relationship between the decrease in survival and the positivity of cerb-B2, it should be noted that survival-related outcomes are controversial. Much work has been done on this subject [12-13]. Slamon et al. showed a significant independent relationship between cerb-B2 positivity and recurrence (p=0.001) and poor survival (p=0.02) in a multivariate analysis of the axillary negative case group [12]. There are also studies showing that cerb-B2 positivity refers to decreased survival in patients with axillary positive [14]. In our study, in cases with cerb-B2 positive, a statistically significant difference was found between the dead cases and the stage distributions of the right cases. Since no death was observed in the cerb-B2 negative patients, no statistical evaluation was performed. However, compared to the rate of death compared to the stages. In Stage 2 and Stage 3 cases, cerb-B2 positivity shows decreased survival.

## Conclusions

As a result, all breast cancer patients should be staged preoperatively and postoperatively, factors affecting the prognosis of patients should be determined, and treatment protocols specific to each patient should be determined. Axillary lymph node involvement and number, tumor size, hormone receptor status, and cerb-B2 status are safe markers that can be used to determine the prognosis of patients.
